# Activity monitoring and patient-reported outcome measures in Myalgic Encephalomyelitis/Chronic Fatigue Syndrome patients

**DOI:** 10.1371/journal.pone.0274472

**Published:** 2022-09-19

**Authors:** Ingrid G. Rekeland, Kari Sørland, Ove Bruland, Kristin Risa, Kine Alme, Olav Dahl, Karl J. Tronstad, Olav Mella, Øystein Fluge

**Affiliations:** 1 Department of Oncology and Medical Physics, Haukeland University Hospital, Bergen, Norway; 2 Department of Clinical Sciences, University of Bergen, Bergen, Norway; 3 Department of Medical Genetics, Haukeland University Hospital, Bergen, Norway; 4 Department of Biomedicine, University of Bergen, Bergen, Norway; Prince Sattam Bin Abdulaziz University, College of Applied Medical Sciences, SAUDI ARABIA

## Abstract

**Introduction:**

Myalgic Encephalomyelitis/Chronic Fatigue Syndrome (ME/CFS) is a disease with no validated specific and sensitive biomarker, and no standard approved treatment. In this observational study with no intervention, participants used a Fitbit activity tracker. The aims were to explore natural symptom variation, feasibility of continuous activity monitoring, and to compare activity data with patient reported outcome measures (PROMs).

**Materials and methods:**

In this pilot study, 27 patients with mild to severe ME/CFS, of mean age 42.3 years, used the Fitbit Charge 3 continuously for six months. Patients wore a SenseWear activity bracelet for 7 days at baseline, at 3 and 6 months. At baseline and follow-up they completed the Short Form 36 Health Survey (SF-36) and the DePaul Symptom Questionnaire–Short Form (DSQ-SF).

**Results:**

The mean number of steps per day decreased with increasing ME/CFS severity; mild 5566, moderate 4991 and severe 1998. The day-by-day variation was mean 47% (range 25%–79%). Mean steps per day increased from the first to the second three-month period, 4341 vs 4781 steps, p = 0.022. The maximum differences in outcome measures between 4-week periods (highest vs lowest), were more evident in a group of eight patients with milder disease (baseline SF-36 PF > 50 or DSQ-SF < 55) as compared to 19 patients with higher symptom burden (SF-36 PF < 50 and DSQ-SF > 55), for SF-36 PF raw scores: 16.9 vs 3.4 points, and for steps per day: 958 versus 479 steps. The correlations between steps per day and self-reported SF-36 Physical function, SF-36 Social function, and DSQ-SF were significant. Fitbit recorded significantly higher number of steps than SenseWear. Resting heart rates were stable during six months.

**Conclusion:**

Continuous activity registration with Fitbit Charge 3 trackers is feasible and useful in studies with ME/CFS patients to monitor steps and resting heart rate, in addition to self-reported outcome measures.

**Clinical trial registration:**

Clinicaltrials.gov: NCT04195815.

## 1. Introduction

Myalgic Encephalomyelitis/Chronic Fatigue Syndrome (ME/CFS) is a disease of unknown etiology with high symptom burden, no validated specific and sensitive biomarker, and no standard approved effective treatment. Defined by the Canadian consensus criteria, it affects 0.1–0.8% of the population [1, 2]. ME/CFS has a profound impact on the quality of life of both patients and caregivers, and entails high costs for society [3, 4].

Our working hypothesis is that ME/CFS in a subgroup could be a variant of an autoimmune disease. We have previously conducted clinical trials in patients with ME/CFS using the immunomodulatory drugs cyclophosphamide and rituximab [5–8]. In these studies, primary and secondary endpoints were based on various questionnaires for patient-reported outcome measures (PROMs) such as symptom change during follow-up and health-related quality of life. Activity monitoring using Sensewear armband were also used for secondary endpoints (mean steps per 24 hours); however, these armbands, although validated for clinical studies, had technical limitations and were used to record data for no more than 7 consecutive days at a time.

In the randomized and placebo-controlled trial assessing rituximab induction and maintenance infusions versus placebo in ME/CFS patients, the primary and secondary outcome measures were negative with no significant differences between the rituximab and placebo groups [7]. There were, however, fluctuations and changes in symptoms and activity level in both groups through follow-up. From our experience of these trials, we believe that there is a need for better knowledge and characterization of symptom variation over time in ME/CFS, independently of any treatment or intervention. An improved understanding of the natural course of ME/CFS over time would aid the development of better outcome measures and endpoints for future clinical trials, and contribute to distinguish natural symptom variation from changes in disease course which could be ascribed to trial intervention. Also, there is a need for objective parameters describing aspects of the ME/CFS disease, in addition to questionnaires designed to capture subjective and self-reported data.

In addition, comparison of data from different clinical trials are hampered by the use of different outcome measures and, importantly, inclusion of patient populations with different levels of symptom severity. The establishment of new, improved and broadly accepted outcome measures would aid such comparisons, which we believe are important when assessing trial outcomes. Until now, consensus on outcome measures is lacking, but there are some reports evaluating different PROMs in ME/CFS [9–15]. Several studies report data from the self-reported questionnaire for health-related quality of life Short Form 36 Health Survey (SF-36), with focus on the subscale Physical Function (SF-36 PF), and the DePaul symptom questionnaire (DSQ).

Aiming to improve the outcome measures for future clinical trials on ME/CFS, this study evaluates the use of data obtainable from activity trackers, which are growing in popularity. Several studies for evaluation of accuracy have been performed [16]. Both our group and others have used steps per 24 hours as an objective outcome measure in studies with ME/CFS, using different activity tracker technologies [5–8, 17–19]. The overall impression is that the measurement of steps in particular seems to have acceptable accuracy in controlled studies, and may be a useful readout in a clinical setting [16, 20, 21].

In this prospective observational study with no intervention, 27 participants used Fitbit activity trackers continuously throughout the six-month study period. We explored the feasibility of continuous activity monitoring in a clinical trial and whether the armbands could be used to assess levels of physical activity in ME/CFS patients. Further, we compared the continuous activity monitoring with PROMs for health-related quality of life and for ME/CFS symptoms, and asked the participants to assess which data best reflected their own perception of activity level and symptom severity. Data from the Fitbit monitor were compared with the SenseWear activity armbands, which have been validated and used in our previous clinical trials.

## 2. Materials and methods

### 2.1 Trial design

The study (ClinicalTrials.gov NCT04195815) was designed as a prospective observational study with continuous monitoring of physical activity using Fitbit Charge 3 activity trackers for 6 months, including assessment of feasibility, comparison with PROMs for quality of life (SF-36 ver.1.2) and for ME/CFS symptoms (DSQ-SF), and comparison with the validated activity bracelet Sensewear.

### 2.2 Setting and patient inclusion

Recruitment was performed by advertising via the local ME association’s Facebook page, and the research group’s e-mail newsletter. Of 122 candidates who applied for participation, 14 were excluded due to long travel distance to the study center, and 16 because they had participated in earlier studies. From the remaining 92 candidates, 30 were randomly selected and informed about the study by phone. Three patients decided not to participate, and the remaining 27 proceeded to clinical assessment and inclusion. Due to the Covid-19 pandemic, the inclusion process was closed after the first 27 included patients.

Inclusion criteria were: a diagnosis of ME/CFS according to the Canadian consensus criteria [22]; age 18 to 65 years; disease duration more than two years; and disease severity mild, mild-to-moderate, moderate, moderate-to-severe, or severe. For statistical analyses, these were lumped into three categories: mild (including mild and mild-to-moderate), moderate, and severe (including moderate-to-severe and severe). The exclusion criteria and baseline clinical evaluation with laboratory tests are detailed in the trial protocol (S1 File).

Recruitment and follow-up lasted from March 2020 until November 2020. All 27 patients were included and monitored at the Department of Oncology and Medical Physics, Haukeland University Hospital (HUH).

### 2.3 Activity armbands and data collection

The patients used Fitbit Charge 3 trackers on their non-dominant wrist (Fitbit, San Francisco, US) for continuous monitoring of physical activity, day and night. They were instructed to only remove the trackers for recharging, roughly for one hour per week.

A Data Protection Impact Assessment was performed prior to study start. In an effort to protect the participants’ privacy, we used pseudonymisation toward third parties. Each participant Fitbit account was set up using a study-specific e-mail address, initials instead of name and a fictitious date of birth. Fitbit’s terms of use comply with the General Data Protection Regulation (GDPR) directive. Fitbit activity data from each participant were downloaded at the study center weekly, using the Fitbit web API (https://dev.fitbit.com/build/reference/web-api/developer-guide/application-design/). For each participant we registered an Oauth 2.0 application with type set as “personal”. The scopes were set to heartrate+profile+sleep+settings+activity+weight, with “time” set to 365 days.

An R-script was generated to facilitate downloading of data from all participants (S2 File). For more detailed information on data protection issues see trial protocol (S1 File).

In order to compare Fitbit activity data to the SenseWear device used in our previous studies, patients were instructed to wear a Sensewear armband on the non-dominant upper arm, continuously for 7 days, at baseline and two time points during follow-up (at 3 and 6 months). Sensewear armbands have been validated for use in clinical studies [23, 24].

During recent years, several clinical studies for validation of older generations of Fitbit trackers, in different diseases, have also been performed. The overall impression is that from the different measures available, number of steps had the best accuracy [16, 20, 21]. Sleep measures, energy expenditure and heart rate in the higher heart rate zones were found to be less accurate [25, 26]. In this study we focused on the measured “steps per 24 hours” and “resting heart rate” recorded during sleep or at inactivity during the day.

### 2.4 Self-reported questionnaires

At baseline, the patients recorded self-reported symptom score for Fatigue, PEM, and need for rest using a scale of 1 to 10 (higher number denotes more severe symptoms), and Function level (scale 1 to 100%) according to a table with examples in which 100% was completely healthy (S1 File).

At baseline and every four weeks, they completed Norwegian-language versions of the SF-36 questionnaire for health-related quality of life (Short Form 36 Health Survey ver. 1.2) [27], and the DePaul Symptom Questionnaire—Short Form (DSQ-SF) for ME/CFS symptoms [15], Norwegian translation of DSQ-SF is based on the translation of the complete DePaul Symptom Questionnaire [28]. (English versions in S1 File).

During follow-up, patients completed the Composite Autonomic Symptom Score-31 (COMPASS-31) questionnaire, used to assess symptoms related to dysautonomia.

One week after completing the study, the participants were asked to answer an evaluation of the study and the activity armband. We used an online survey from enalyzer.com. The answers were anonymous.

### 2.5 Statistics

For Fitbit data, steps per 24 hours and resting heart rate, with continuous data for 168 days (24 weeks), with no replacement for missing data, were used in the analyses. Means and standard deviations (SD) were calculated per 4-week period, and also dichotomized into days 1–84 vs days 85–168, to assess changes over time. Groups were compared by t-test (equal variance not assumed), or by Mann-Whitney test, as appropriate.

Repeated measures of variables, by ME/CFS severity and by categories of baseline SF-36 PF, were assessed by General Linear Model (GLM) for repeated measures.

Correlations (Spearman’s rho) were performed between different variables describing aspects of ME/CFS such as Function (%), SF-36 domains, Compass sum score and domains, DSQ-SF total score, mean steps and resting HR.

All tests were two-sided with a significance level of p < 0.05. Missing data were replaced only for one patient at the 24-weeks recording for SF-36 and DFQ-SF (i.e. 2 recordings out of 378) using the last value carried forward (LVCF) method. All analyses were performed using IBM SPSS Statistics ver.26 (IBM Corp., Armonk, USA), and Graphpad Prism ver.9 (GraphPad Software, La Jolla, USA).

### 2.6 Ethics

The study was approved by the Regional Committees for Medical and Health Research Ethics in Norway (No. 28780). Concerns about patients’ privacy are discussed under “Activity armbands” in Methods. A Data Protection Impact Assessment was performed in consultation with the IT Security Manager and Data Protection Officer for the Bergen Hospital Trust and user representatives. The user representatives have also assessed and contributed to the study protocol and all written information given to the participants. All participants gave written consent.

## 3. Results

### 3.1 Study population

**Fig 1:**
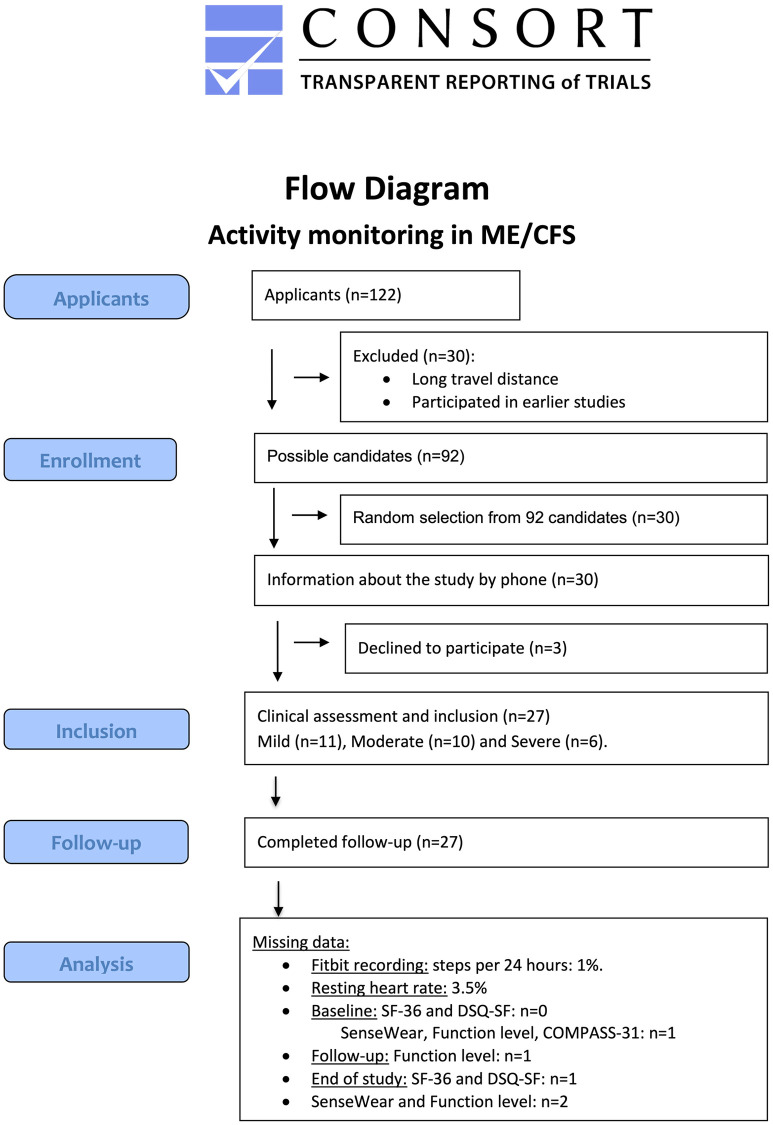
CONSORT flow diagram. Flow diagram of enrollment, follow-up and analysis in the study “Activity monitoring in ME/CFS”.

Flow diagram of participants through enrollment, inclusion and follow-up and analysis are shown in Fig 1. After prescreening (described in Methods), 27 patients were included. All participants had an established ME/CFS diagnosis and met Canadian inclusion criteria by clinical assessment. Patients with a relatively mild degree of ME/CFS symptoms were somewhat overrepresented; this was accepted because the main purpose of the study was to assess the feasibility and usefulness of continuous activity monitoring and PROMs, and a representative sample was not required. Based on clinical assessment, participants were divided into three severity categories; mild (n = 11), moderate (n = 10) and severe (n = 6). Table 1 shows baseline characteristics for all included patients (n = 27), and also by severity group.

**Table 1:** Baseline characteristics of the study population, all patients and by severity.

Characteristic	All patients (n = 27)	Mild (n = 11)	Moderate (n = 10)	Severe (n = 6)	P-value (ANOVA)	P-value (Trend)^9^
Female, n	25	10	9	6	-	-
Male, n	2	1	1	0	-	-
Age, all patients, mean (min-max)	42.3 (20–62)	40.6 (20–58)	44.3 (20–62)	42 (31–60)	0.75	0.72
BMI^1^ all patients, mean (min-max)	28.0 (20.0–44.0)	26.5 (21.4–37.6)	31.1 (20.6–44.0)	27.6 (21.5–31.7)	0.43	0.57
Systolic blood pressure, mean (min-max)	132 (104–170)	128 (104–154)	143 (122–170)	122 (107–141)	0.04	0.86
Diastolic blood pressure, mean, (min-max)	85 (63–105)	81 (63–94)	92 (81–105)	82 (66–101)	0.07	0.49
Mean resting HR^2^	68.5 (55–95)	67 (55–80)	71 (61–95)	65 (56–71)	0.40	0.55
*ME/CFS disease duration*						
2–5 years, n	6	4	2	0	-	
5–10 years, n	8	3	3	2	-	
10–15 years, n	7	1	4	2	-	
>15 years	6	3	1	2	-	
Self-reported Fatigue^3^, mean (min-max)	7.1 (5–9)	6.4 (5–8)	7 (6–8)	8.5 (7–9)	<0.001	<0.001
Self-reported post-exertional^3^ malaise, mean (min-max)	7.7 (6–10)	7.1 (6–9)	7.6 (7–8)	8.8 (8–10)	0.001	<0.001
Need for rest^3^, mean (min-max)	7.2 (5–9)	6.5 (5–8)	7.4 (7–8)	8.2 (8–9)	<0.001	<0.001
Function level^4^, mean (min-max)	18.0 (5–35)	26.4 (20–35)	16.2 (10–20)	8.0 (5–12)	<0.001	<0.001
SF-36^5^ Physical Function, mean (min-max)	37.2 (5–70)	46.4 (25–70)	35.5 (25–65)	23.3 (5–45)	0.024	<0.01
SF-36^5^ Bodily pain, mean (min-max)	38.3 (0–84)	44.7 (22–84)	39.4 (22–74)	24.7 (0–41)	0.093	0.04
SF-36^5^ General Health, mean (min-max)	32.3 (10–65)	36.1 (15–65)	33.0 (10–50)	24.2 (10–45)	0.297	0.14
SF-36^5^ Vitality, mean (min-max)	23.9 (0–70)	18.2 (0–40)	27.5 (10–35)	28.3 (10–70)	0.257	0.14
SF-36^5^ Social Function, mean (min-max)	28.2 (0–75)	37.5 (13–75)	30.0 (0–75)	8.3 (0–25)	0.018	<0.01
SF-36^5^ Mental health, mean (min-max)	78.4 (44–92)	76.0 (56–92)	81.6 (44–92)	77.3 (64–88)	0.600	0.7
DSQ-SF^6^ total score, mean (min-max)	67.2 (38–106)	62.9 (42–87)	62.8 (38–81)	82.3 (63–106)	0.022	0.02
Steps per 24 hours^7^, Fitbit mean (min-max)	4305 (756–8541)	5007 (3756–8199)	4927 (2895–8541)	1979 (756–4056)	0.001	0.02
Compass-31^8^ sum score, mean (min-max)	41.9 13.3–63.7	36.8 13.3–54.9	42.5 33.5–57.6	49.4 38.2–63.7	0.114	0.04
Compass orthostatic^8^, mean (min-max)	19.2 0–28.0	15.0 0–24.0	20.0 12.0–28.0	24.0 16.0-28-0	0.073	0.02

^1^Body Mass Index;

^2^Mean resting heartrate weeks 1–4,

^3^Self reported scale from 1–10 (higher number denotes more symptoms)

^4^Self-reported scale 1–100 (higher number denotes better function), according to a table with examples

^5^Short Form-36 Health Survey (SF-36) with raw scores (scale 0–100). Higher number denotes less symptoms.

^6^DePaul Symptom Questionnaire–Short Form (DSQ-SF), higher number denotes more symptoms.

^7^Steps mean per 24 hours, week 0–4.

^8^Composite Autonomic Symptom Score-31 (COMPASS-31); sum score and the domain “Compass orthostatic”, higher number denotes more symptoms.

^9^ANOVA trend test

Missing data for one patient for Blood Pressure, self-reported Fatigue, PEM, Need for rest, Steps and Compass score.

Except for one participant who started a low carbohydrate diet aimed at the ME/CFS symptoms, the participants did not undergo any intervention for their ME/CFS disease during the course of the study.

A dataset with clinical data, PROMS, steps and resting heartrate for all patients during follow up (S3 File) and TREND checklist for clinical studies (S4 File) are found in supplementary files.

### 3.3 Missing data

There were little missing data from the Fitbit recordings of activity level. For the 24-week study period, the mean number of days with valid recording of steps per 24 hours was 166 out of 168 days. There were recordings of steps per 24 hours for all 168 days in 20 patients, while 2 had recordings from 167 days, and 5 patients had valid recordings spanning from 152 to 166 days.

For resting heart rate, the mean number of days with valid recordings was 162. Sexteen patients had recordings from at least 166 days, and 11 patients had valid recordings spanning from 132 to 165 days. For steps per 24 hours and resting heart rate, the observed data were used as input for statistical analyses, with no replacement for missing values (approximately 0,1% and 0,5% missing data for steps per 24 hours and for resting heart rate, respectively).

At baseline there were no missing data for SF-36 and DSQ-SF, and only missing data from one patient for SenseWear, Function level and COMPASS-31. During follow-up one patient failed to report Function level, and at 24 weeks (end of study) there were missing data for SenseWear and Function level for two patients, the observed data were used with no replacement for missing values.

For SF-36 and DSQ-SF one patient had missing data at the 24-week recording (i.e. 2 missing out of 378 recordings, 0.5%). These two data for SF-36 and DSQ-SF were replaced by the LVCF method.

### 3.4 Fitbit; steps and resting heart rate

**Fig 2:**
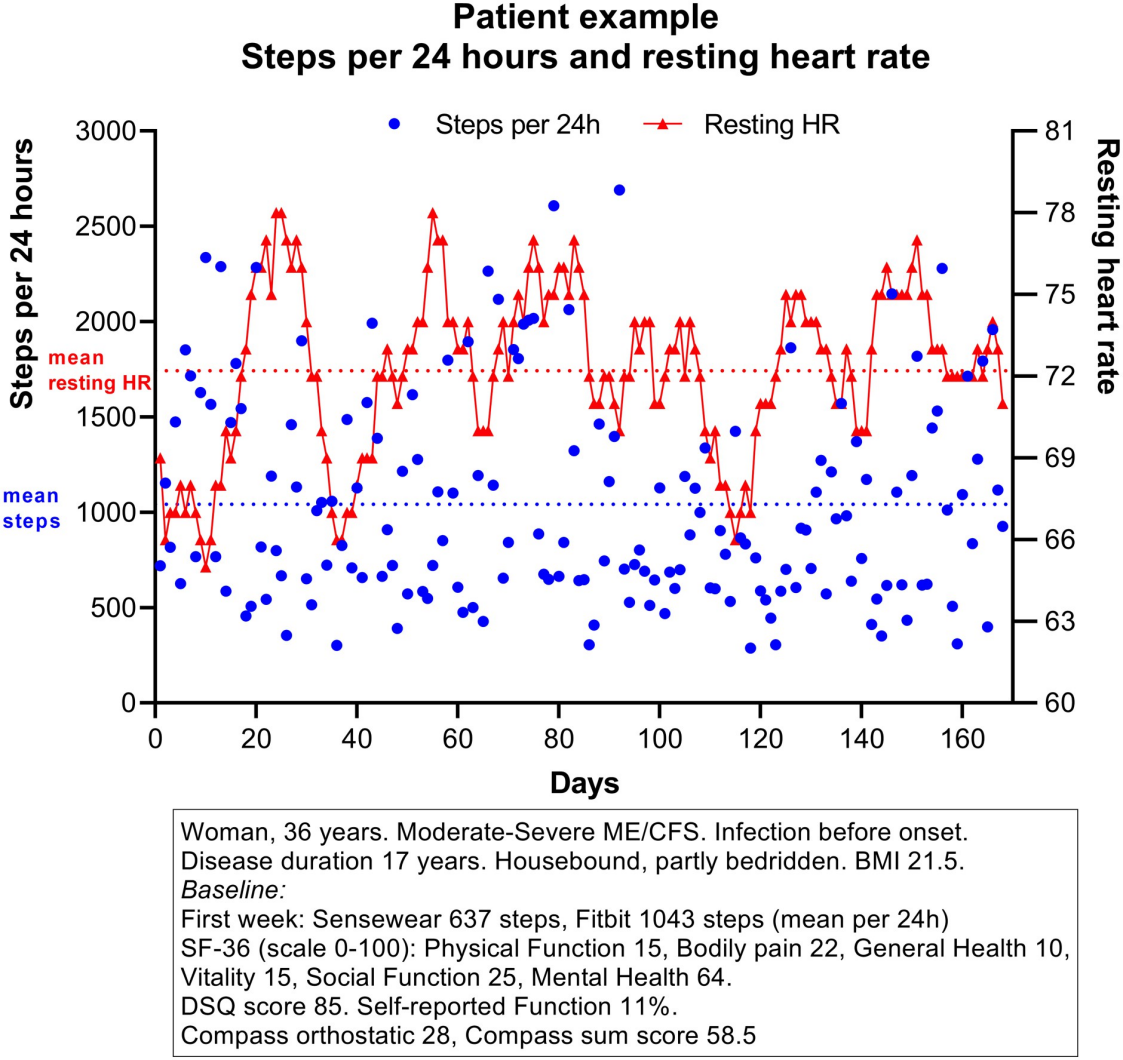
Patient example. Patient example with raw data for steps per 24 hours and resting heart rate, days 1–168.

**Fig 3:**
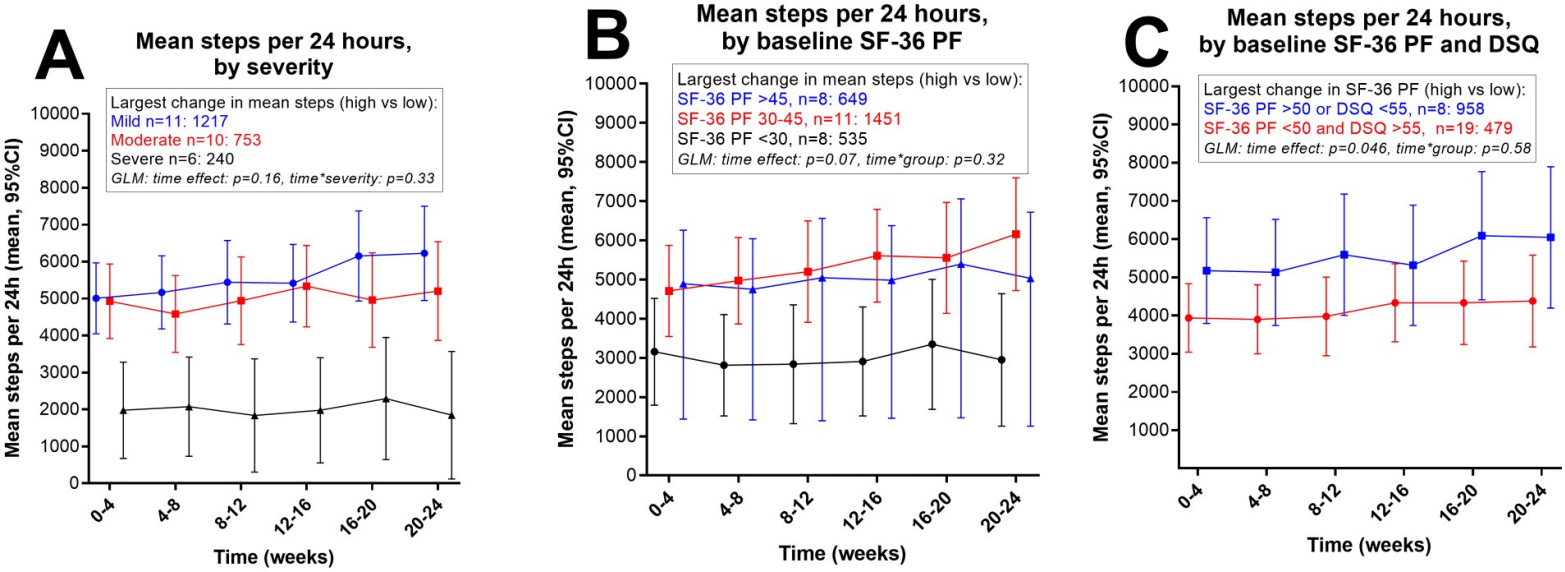
Steps per 24 hours, by severity, by SF-36 physical function, and by combination of SF-36 physical function and DSQ-SF. (A) Steps per 24 hours (mean, 95% CI) during follow-up, by severity categories; Mild, Moderate and Severe. (B) Steps per 24 hours (mean, 95% CI) during follow-up, by three categories based on baseline SF-36 PF. (C) Steps per 24 hours (mean, 95% CI) during follow-up, in two groups based on; SF-36 PF > 50 or DSQ-SF < 55, versus SF-36 PF < 50 and DSQ-SF > 55. The largest changes in mean steps between 4-week time periods, with difference highest versus lowest are indicated. General Linear Model (GLM) for repeated measures with p values for time effect and for interaction time-by-group are shown.

Mean steps per 24 hours for all 27 patients during 168 days’ follow-up were 4560. A representative profile for steps per 24 hours and resting HR throughout the study period is shown in Fig 2. The mean number of steps per 24 hours decreased with increasing severity with a significant ANOVA trend test; mild 5566 steps, moderate 4991 steps and severe 1998 steps (p = 0.02). The variation in mean steps per 24 hours (i.e. the difference between 4-week periods with highest and lowest values), were 1217 steps among patients with mild severity, 753 in moderate, and 240 in severe ME/CFS (Fig 3A). Baseline steps (week 0–4) for all patients and for the three severity groups are shown in Table 1.

For day-by day variation of steps per 24 hours, the mean coefficient of variation (CV, defined as SD/mean) was 47%, however with a broad range 25%–79% among patients, and with no significant difference between clinical severities, or between categories of SF-36 PF.

**Fig 4:**
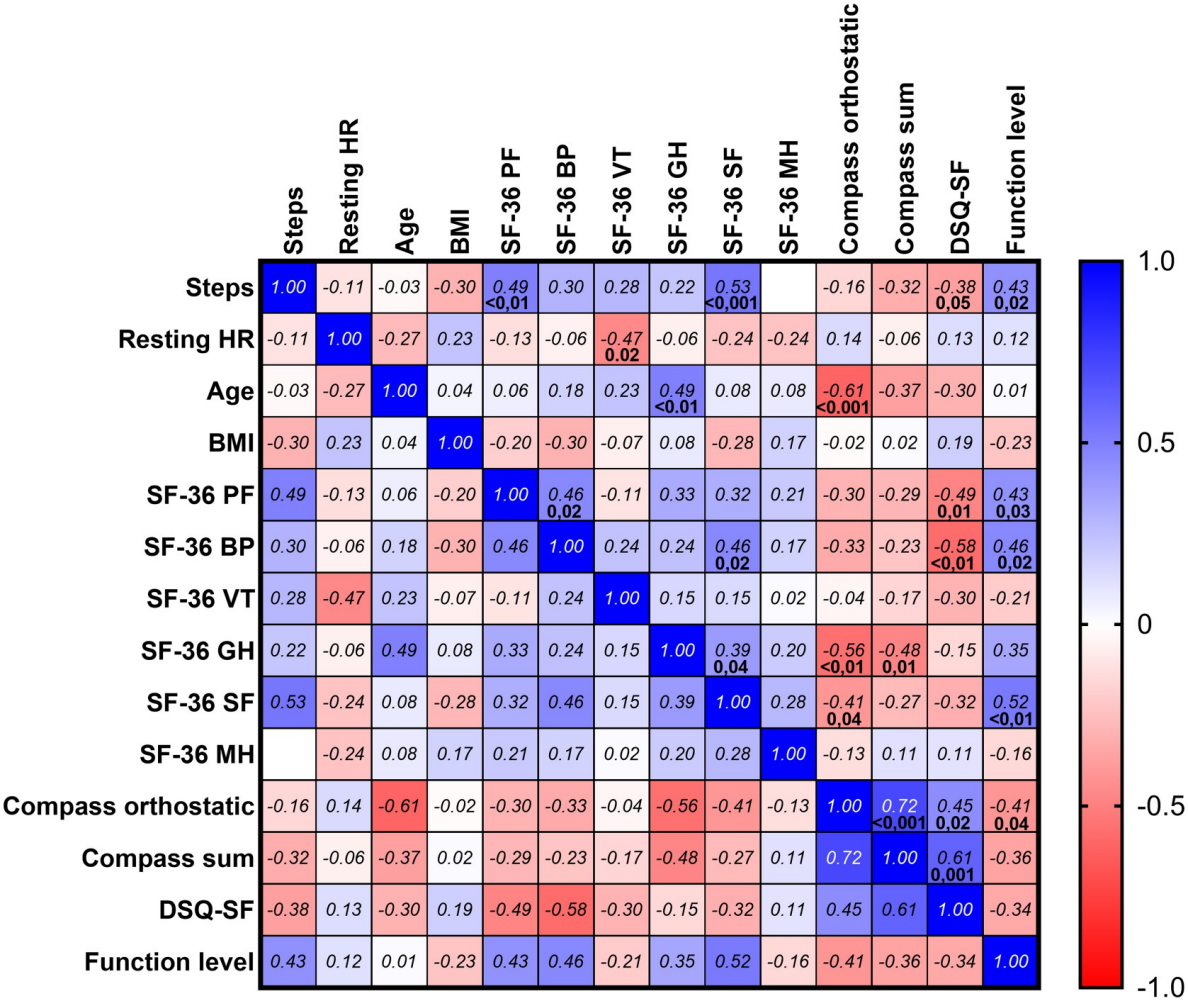
Correlations between baseline clinical data, PROMs, steps per 24 hours and resting heart rate. Spearman’s correlation plot between baseline steps per 24 hour (mean, weeks 1–4), resting heart rate (mean, weeks 1–4), age, Body Mass Index. Short Form-36 Health Survey (SF-36); The raw scores (scale 0–100) for the six SF-36 domains (Mental health (SF36-MH), Physical function (SF-36 PF), Bodily pain (SF-36 BP), General health (SF-36 GH), Social function (SF-36 SF) and Vitality (SF-36 VF). Composite Autonomic Symptom Score-31 (COMPASS-31); sum and Compass orthostatic, DePaul Symptom Questionnaire–Short Form (DSQ-SF), and Function Level. Significant p-values are shown below Spearman’s rho, with no adjustments for multiple correlations.

Mean steps per 24 hours for all 27 patients through days 1–84 compared to days 85–168, assessed by paired sample t-test, was significantly higher in the last period (4341 vs 4781 steps, p = 0.022). The absolute changes in mean steps per 24 hours (with minimum and maximum) from the first to the second half of the study were mean 723 steps (-960 to 4031) in patients with mild disease, mean 350 steps (-415 to 1236) in patients with moderate severity, and mean 68 (-263 to 291) in patients with severe ME/CFS. Although the increase in mean steps per day through follow-up was largest among patients with mild severity, or with higher baseline SF-36 PF, the changes were not significantly different by ME/CFS severity, (Fig 3A), or by categories of baseline SF-36 PF (Fig 3B) during follow up, assessed by GLM repeated measures. At baseline, mean steps per 24 hours (recorded weeks 1–4) correlated significantly with SF-36 PF (p = 0.01 by Spearman’s rho) (Fig 4).

**Fig 5:**
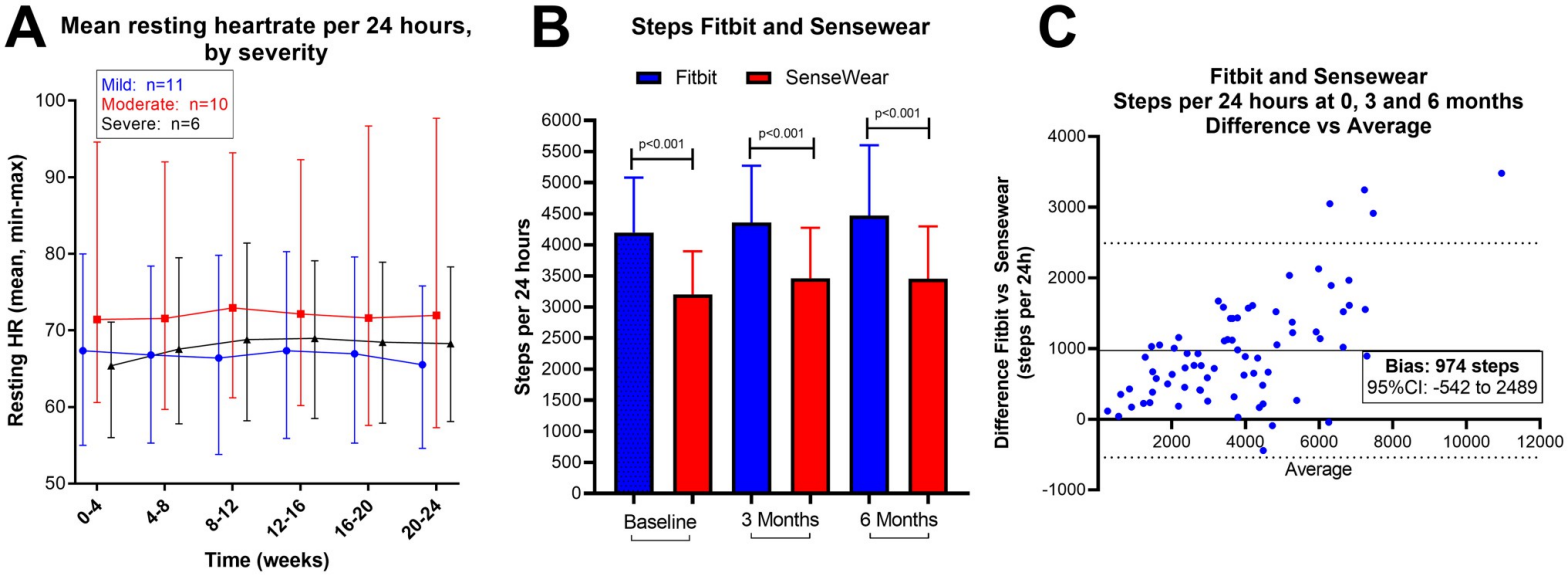
Activity data: Resting heart rate by severity, steps per 24 hours measured with Fitbit and SenseWear. (A) Resting heart rate, mean (min and max) levels, by three severity groups. (B) Steps per 24 hours measured for seven consecutive days by Fitbit and SenseWear, at baseline, 3 months and 6 months. (C) Bland-Altman plot showing difference (bias) between Fitbit and SenseWear devices for measured steps per 24 hours.

Mean resting heart rates were stable during follow-up in the three severity groups, and also by categories of baseline SF-36 PF. Correlation between steps per 24 hours and resting heart rate (recordings days 1–168) was not significant (p = 0.58). Note the considerable individual variation between minimum and maximum mean resting heartrate (Fig 5A).

A dataset with mean steps and resting heartrate per day for each patient is found in S5 File.

### 3.5 Comparison of Fitbit Charge 3 and SenseWear; steps

Recordings of steps per 24 hours using the Fitbit Charge 3 tracker (on the non-dominant wrist) were compared with recordings from the SenseWear activity device (on the non-dominant upper arm) for seven days at baseline, at 3 months and at 6 months. Values from the two activity trackers correlated significantly at all three timepoints, but Fitbit recorded significantly higher number of steps (Fig 5B). A Bland-Altman plot (Fig 5C) showed a systematic difference between the two devices, with a bias of 974 steps per 24 hours, (95% CI -542 to 2489), which corresponds to a bias of 27.5% (95% CI -5% to 60%).

A dataset with the comparison between Sensewear and Fitbit is found in S6 File.

### 3.6 Self-reported questionnaires for health-related quality of life

**Fig 6:**
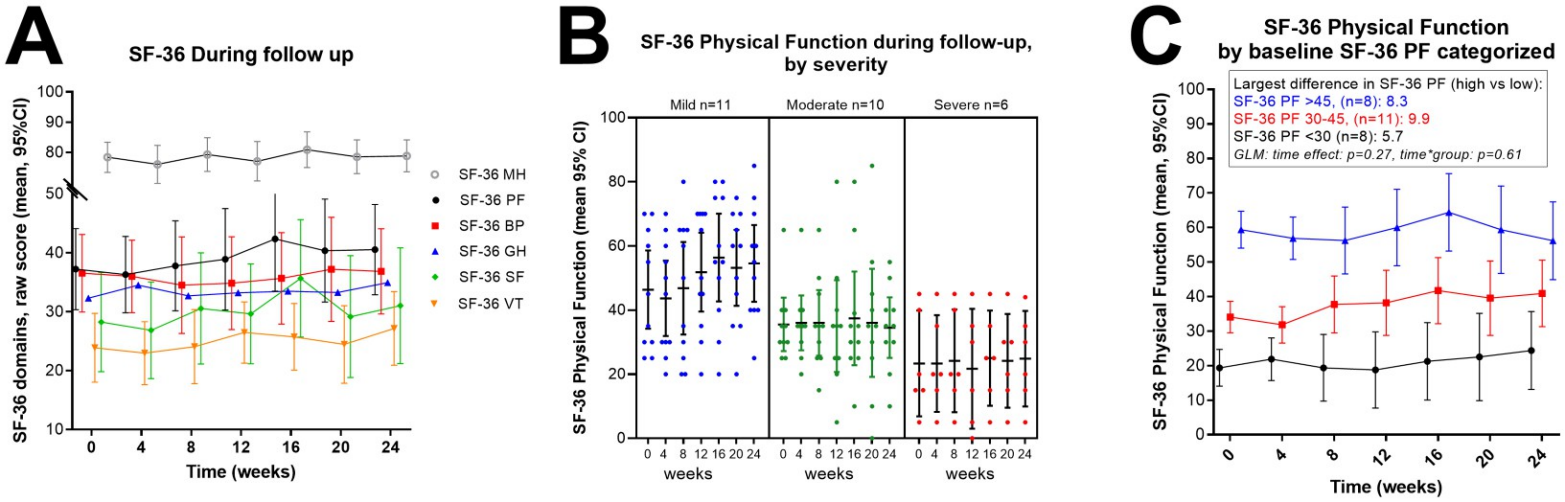
SF-36 subdomains (mean, 95% CI) during follow up; SF36 Physical Function (SF-36 PF) by severity categories, and by categories of baseline SF-36 PF. (A) SF-36 domains during follow-up; MH: Mental Health, PF: Physical Function, BP: Bodily pain, GH: General health, SF: Social function and VT: Vitality. Raw scores, scale 0–100, lower scores denote lower function. (B) SF-36 Physical Function (mean, 95% CI) during follow up shown in separate panels, for the severity categories. (C) SF-36 Physical Function (mean, 95% CI) during follow-up by three categories based on the baseline level of SF-36 PF; < 30, 30–45, and > 45. General Linear Model (GLM) for repeated measures with p values for time effect and for interaction time-by-group are shown.

The raw scores (scale 0–100) for the six SF-36 domains (Mental health (SF-36 MH), Physical function (SF-36 PF), Bodily pain (SF-36 BP), General health (SF-36 GH), Social function (SF-36 SF) and Vitality (SF-36 VF), recorded at four-week intervals during follow-up, are shown in Fig 6A. The Mental health score was stable during follow-up, mean 78.4 (min 73.4—max 83.4) and close to reported values for women in the general population, i.e. mean 79.9 (SD 14.8) [29]. The five other SF-36 domains showed low scores, with the lowest reported for Vitality. Baseline values for all six SF-36 domains, for all patients and divided by the three severity groups are shown in Table 1. Fig 6B shows SF-36 PF in three severity categories during follow-up, and Fig 6C SF-36 PF by categories of baseline SF-36 PF (<30, 30–45, >45).

Mean steps per 24 hours correlated significantly with baseline SF-36 PF (p = 0.01), SF-36 SF (p = 0.03), and baseline DSQ-SF score (p = 0.007). DSQ-SF score correlated significantly with SF-36 PF, SF-36 BP, Function level and Compass sum score. All baseline correlations are shown in Fig 4.

**Fig 7:**
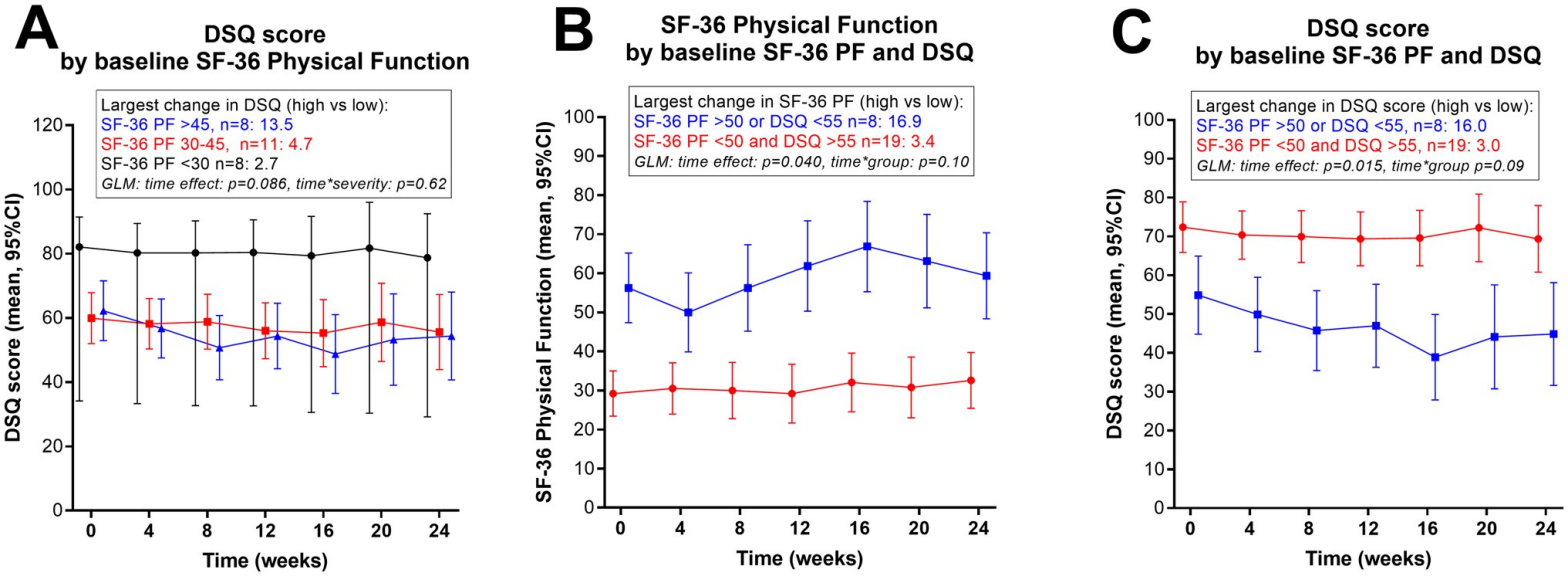
DSQ-SF and SF-36 Physical Function during follow-up, by ME/CFS categorized based on a combination of baseline SF-36 PF and DSQ-SF scores. (A) DePaul Symptom Questionnaire–Short Form (DSQ-SF) score (mean, 95% CI) during follow-up, by categories based on baseline SF-36 Physical Function; < 30, 30–45, and > 45. (B) SF-36 Physical function score (mean, 95% CI) during follow-up, by two groups based on: SF-36 PF > 50 OR DSQ-SF < 55 versus SF-36 PF < 50 AND DSQ-SF > 55. (C) DSQ-SF score (mean, 95% CI), by two groups based on; SF-36 Physical Function >50 OR DSQ-SF < 55 versus SF-36 Physical Function < 50 AND DSQ-SF > 55. The largest changes in mean scores between 4-week time periods, with difference highest versus lowest, are indicated. General Linear Model (GLM) for repeated measures with p values for time effect and for interaction time-by-group are shown.

Baseline DSQ-SF scores for ME/CFS symptoms are reported in Table 1. The mean scores for the mild and moderate groups were similar, but significantly higher for the severe group. Fig 7A shows DSQ-SF scores during follow-up categorized by baseline SF-36 PF.

In an explorative attempt to define groups of patients with the largest variations in outcome measures during six months’ follow-up, we combined baseline SF-36 PF and DSQ-SF scores. One group of 8 patients with milder ME/CFS symptoms had either SF-36 PF > 50 or DSQ-SF < 55, while the remaining group of 19 patients with more pronounced ME/CFS symptoms had both SF-36 PF < 50 and DSQ-SF > 55. In this observational study with no intervention, the maximum differences between each 4-week period were more evident in the group of patients with milder ME/CFS, for mean number of steps per 24 hours (mean increase of 958 steps versus 479 steps, Fig 3C), for SF-36 PF scores (16.9 vs 3.4 points, Fig 7B), and for DSQ-SF (16.0 vs 3.0 points, Fig 7C).

### 3.7 Compass-31

During follow-up the patients answered the questionnaire COMPASS-31 to map symptoms of autonomic dysfunction. COMPASS sum score and the domain “orthostatic intolerance” are shown in Table 1.

There were significant positive associations between the sum score of COMPASS-31 and of the domain “orthostatic intolerance,” and the three levels of ME/CFS severity (p = 0.037 and p = 0.035, respectively). There were significant negative correlations between “Orthostatic intolerance” and the patient’s age, the SF-36 domain “General health” and Function level (Fig 4).

### 3.8 Participant evaluation of the study

One week after completing the study the participants were asked to answer an evaluation of the study and the activity armband. We used an online survey from enalyzer.com. The answers were anonymous and 22 of the 27 participants answered (Table 2).

**Table 2:** Participant evaluation of the study.

Question	Disagree^1^N, (%)	Agree^2^ N, (%)	Undecided^3^ N, (%)
**Fitbit armband n = 23**			
Fitbit app was easy to use	3 (13)	20 (87)	0
The Fitbit armband was comfortable to use	1 (4)	22 (96)	0
I experienced discomfort by using Fitbit armband	17 (74)	5 (22)	1 (4)
I had problems with the armband or the app, that I could not solve by simple troubleshooting	17 (74)	5 (22)	1 (4)
Using Fitbit affected my activity level	11 (48)	9 (39)	3 (13)
I used Fitbit as a tool to regulate my activity level	5 (22)	10 (44)	8 (35)
**In my experience, the following measures reflected my activity level: n = 23**			
Steps	4 (17)	17 (74)	2 (9)
Heartrate	0	22 (96)	1 (4)
Sleep	8 (35)	10 (44)	5 (22)
Active minutes	4 (17)	10 (44)	9 (39)
**SF-36 n = 22**			
The questionnaire was difficult to complete	16 (73)	5 (23)	1 (4)
Completing the questionnaire took a lot of effort	12 (54)	7 (32)	3 (14)
The questions were easy to understand	5 (23)	15 (68)	2 (9)
The questions were relevant for my situation	2 (9)	18 (82)	2 (9)
The questionnaire captured the changes in my condition	4 (18)	13 (59)	5 (23)
**DePaul Symptom Questionnaire—Short Form n = 22**			
The survey was difficult to answer	14 (64)	2 (9)	6 (27)
I used a lot of effort to answer * n = 23?	13 (57)	6 (26)	4 (17)
The questions were easy to understand	2 (9)	17 (77)	3 (14)
The questions were relevant for my situation	1 (4)	18 (82)	3 (14)
The survey captured the changes in my condition	2 (9)	14 (64)	6 (27)
**Self-reported symptom change every two weeks n = 22**			
The survey was difficult to answer	20 (91)	1 (5)	1 (5)
I used a lot of effort to answer	19 (86)	1 (5)	2 (9)
The questions were easy to understand	0	20 (91)	2 (9)
The questions were relevant for my situation	0	20 (91)	2 (9)
The survey captured the changes in my condition	1 (5)	17 (77)	4 (18)

^1^The column “Disagree” contains three levels of disagree; totally-, quite- and slightly disagree.

^2^The column “Agree” contains of three levels of agree; totally-, quite- and slightly agree.

^3^The column “Undecided” contains of “do not know” and “neither nor”.

Several patients reported that their Fitbit devices recorded steps when they were not walking, but engaged in other activites which involved arm movement or vibration, such as knitting, cooking and driving slowly in a car or electric wheelchair on bumpy roads.

## 4. Discussion

The present study shows that continuous activity registration with Fitbit Charge 3 trackers is feasible in studies with ME/CFS patients. The mean number of steps per day decreased with increasing severity. Mean steps per day increased in the second as compared to the first half of the study. The correlations between steps per day and self-reported SF-36 Physical function, Social function, and DSQ-SF were significant. The study had a low number of participants, too few to draw firm conclusions, but using the combination of SF-36 PF and DSQ-SF we identified a group of eight patients with milder disease that showed considerable variation in outcome measures during follow-up compared to the remaining participants.

In attempts to evaluate data from our previous clinical intervention trials of treatment involving the anti-CD20 B-cell depleting antibody rituximab [5–7, 30] or the cytotoxic drug cyclophosphamide [8], we have considered some aspects that may influence trial outcomes and conclusions. These include patient heterogeneity, patient inclusion criteria, case definitions, severity assessment, placebo mechanisms, natural symptom variation over time, and lack of objective outcome measures. There is limited knowledge about the variation and natural course of the ME/CFS disease over time. Moreover, it is not unlikely that patients could be subjected to a trial effect, i.e. the experience of benefit merely by the act of trial participation. In a patient group where the health system generally has little to offer, it is plausible that simply the experience of receiving scheduled follow-up with regular doctor’s appointments during study participation could have some impact on the disease course.

We wanted to evaluate and optimize the possible outcome measures for use in clinical trials by exploring the feasibility of continuous activity monitoring using the Fitbit Charge 3 armband to assess levels of physical activity in ME/CFS patients. Objective measures are important, but in view of the complexity of symptoms involved in this disease, it is necessary to combine the activity measures with self-reported questionnaires. An important question that should be further investigated in larger trials is which technologies and parameters are the most useful to reflect the ME/CFS symptoms and variations over time. Uniformly accepted outcome measures are lacking and criteria for improvement and recovery have been inconsistently defined across studies, making it difficult to assess treatment outcomes and compare different interventional studies.

In recent years, it has been quite common to combine patient-reported questionnaires with activity recordings such as number of steps per day as outcome measures, both in ME/CFS [7, 8, 17–19] and other chronic diseases [20, 21]. Wearable sensors can monitor and detect medical conditions [31], and one study described an alerting system for emerging COVID-19 infection and other stressful events [32]. Advanced studies describe wearable sensors that allow frequent and continuous measurements of different body functions, including HR, skin temperature, blood oxygen levels, physical activity, total gamma and X-ray radiation exposure, and glucose [33]. The use of these devices in the general population is growing in popularity, and many ME/CFS patients already wear some kind of activity device for their personal benefit. The possibilities in the future for wearable sensors in general health and research are vast.

When chosing a device for this project, our priorities were simplicity of use, performance on the basic functionalities (steps and heart rate), and privacy. The Fitbit privacy terms and conditions were more specific on their compliance with the General Data Protection Regulation (GDPR) directive than several comparable trackers in the same price range. Fitbit Charge 3 has been validated and showed acceptable accuracy during rest and treadmill activities, but performed poorly during sprint running and cycling. However, data in the range of activities typical for ME/CFS patients were acceptable [25].

The present study shows that it is feasible to use activity trackers for continuous registration of steps and resting heart rate in a study with ME/CFS patients. Our clinical impression from previous trials, and pilot experiences with ME/CFS patients, was that resting heartrate decreased when patients reported clinical improvement. We did not see the same tendency in this study with no intervention.

Continous Fitbit data for mean steps per 24 hours and for resting heart rate seemed useful, and may be used as outcome measures. However, due to the complexity of symptoms in individual patients, it is still important to also use the patient-reported outcome measures. Patients with ME/CFS cannot be evaluated based exclusively on measures of physical activity.

Generally, both PROMs and number of steps per 24 hours showed slight improvements during six months follow-up. By comparing mean steps per 24 hours in the first 12 weeks versus the last 12 weeks, we found a significant increase in the second part of the study. 23 of 27 patients were included between December and March, which means the first three-month period was winther to spring and the second three-month period was spring to summer. Some of the clinical improvement seen, could be explained by the fact that ME/CFS patients living in the Northern hemisphere often have less severe symptoms when the weather is warm.

The increase in steps and improvements in patient-reported measures were more evident among patients with mild disease. In patients with moderate (mainly housebound) or severe (partly bedridden) ME/CFS, there was little variation in symptom scores or number of steps over time. However, this observation may not be valid for ME/CFS patients in general, due to the low number of patients in the present study. The cause of the increased activity during the study period is not certain. In a recent metaanalysis, feedback through activity monitors was found to increase the daily steps by 1235 in mixed groups of adults [34]. Such an effect could also be relevant for ME/CFS patients, yet with presumably smaller effect sizes due to the debilitating nature of their illness. Improvements in steps per 24 hours and in SF-36 PF scores (increase of 12 points) were also seen during two years follow-up in the placebo-group of the RituxME-trial [7]. We speculate that participation in a study with regular follow-up is in itself beneficial for the patients, and can explain some of the improvements during six months’ follow-up. If the improvements seen in this and other studies can indeed be ascribed to a care effect, this is an important take home message to health services; even if there are limited treatment options available, these patients require qualified and regular support from health care professionals.

When including patients in clinical studies, self-reported medical history and clinical assessments are used for severity grading [35]. A recent study validated the ME/CFS severity by activity bracelets, cardiopulmonary exercise testing and SF-36 [18]. They found that the SF-36 Physical Function subscale (SF-36 PF) and the number of steps per day on an activity meter, showed a clear distinction between mild, moderate and severe ME/CFS patients, with some overlap between the groups. The mean steps by severity groups in this study are in accordance with other studies [17, 18]. For SF-36 PF and steps per day, this distinction between severity groups is similar to what we have seen in our previous studies [7, 8], and also to the present study. The correlation between SF-36 PF and steps was significant, in agreement with a previous study [17].

A systematic review of 56 randomized controlled trials (RCT) for chronic fatigue syndrome/myalgic encephalomyelitis (CFS/ME) showed a total of 31 primary measurement tools used to assess the main outcome. The Checklist Individual Strength (CIS) was the most frequently used (35.7%), and others included the SF-36 (32.1%) [36].

We have used SF-36 in our studies, combined with other self-reported questionnaires. The general experience from our previous studies is that there are overall acceptable agreements between the clinical severity categories, questionnaires such as SF-36 PF and DSQ-SF and steps per day.

When validating and comparing different trials, the characteristics of the included study population are important, and may influence the outcome of the trial. Table 3 shows a selection of studies that have used the SF-36 PF subscale as an outcome measure. The mean baseline SF-36 PF ranged from 15 to 66 in these studies. In the present study, mean SF-36 PF for all patients was 37.2. There is no consensus as to what constitutes a clinically meaningful increase in SF-36 PF. Different studies define various changes in SF-36 PF as response criteria. In the studies described in Table 3, one study used an increase of 25 points in the SF-36 PF as a response criteria [19], while in other studies an increase of 10 [37] or even 7 points [38] was deemed sufficient to signify clinical response. To put this into perspective, the placebo group (n = 74) in our rituximab trial reported a mean increase in SF-36 PF score of 12 points during 24 months’ follow-up (increase from 33 to 45) [7].

**Table 3:** Short Form-36 Health Survey, the domain “Physical Function” (SF-36 PF, raw scores, scale 0–100), in different ME/CFS studies.

Author	Year	Title		SF-36 PF Baseline	SF-36 PF Post intervention
Tummers et al [39]	2010	Effectiveness of Stepped Care (SC) for Chronic Fatigue Syndrome: A Randomized Noninferiority Trial. Care as usual (C)	N = 169 SC, N = 84 C, N = 85	52 (SC) 54 (C)	71 (SC) 72 (C)
White et al [40]	2011	Comparison of adaptive pacing therapy (APT), cognitive behaviour therapy (CBT), graded exercise therapy (GET), and specialist medical care (SMC) for chronic fatigue syndrome (PACE): a randomised trial	N = 641 APT N = 160 CBT N = 161 GET N = 160 SMC N = 160	37 (APT) 39 (CBT) 38 (GET) 39 (SMC)	46 (APT) 58 (CBT) 58 (GET) 51 (SMC)
Tummers et al [41]	2012	Implementing a minimal intervention for chronic fatigue syndrome in a mental health centre: a randomized controlled trial. Guided self-instruction (GSI), Waiting list (WL)	N = 123 GSI, N = 62 WL, N = 61	50 (GSI) 51 (WL)	65 (GSI) 59 (WL)
Fluge et al [6]	2015	B-Lymphocyte Depletion in Myalgic Encephalopathy/ Chronic Fatigue Syndrome. An Open-Label Phase II Study with Rituximab Maintenance Treatment	N = 27	40	67 (at 24 months) 68 (at 36 months)
Pinxsterhuis et al [42]	2017	Effectiveness of a group-based self-management program for people with chronic fatigue syndrome: a randomized controlled trial (I:Intervention, C: Control)	N = 137	46 (I) 46 (C)	48 (I) 51 (C)
Clark et al [43]	2017	Guided graded exercise self-help (GES) plus specialist medical care versus specialist medical care (SMC) alone for chronic fatigue syndrome (GETSET): a pragmatic randomised controlled trial	N = 211 GES, N = 107 SMC, N = 104	47 (GES) 50 (SMC)	56 (GES) 51 (SMC)
Crawley et al [37]	2018	Clinical and cost-effectiveness of the Lightning Process (LP) in addition to specialist medical care (SMC) for paediatric chronic fatigue syndrome: randomised controlled trial	N = 100	56 (SMC) 53 (SMC+LP)	72 (SMC) 86 (SMC+LP)
Stubhaug et al [38]	2018	A 4-Day Mindfulness-Based Cognitive Behavioral Intervention Program for CFS/ME. An Open Study, With 1-Year Follow-Up	N = 305	61 (all pts) 57 (CFS-CDC) 66 (CFS Oxford)	77 (all pts) 75 (CFS-CDC) 76 (CFS Oxford)
Fluge et al [7]	2019	B-Lymphocyte Depletion in Patients With Myalgic Encephalomyelitis/Chronic Fatigue Syndrome: A Randomized, Double-Blind, Placebo-Controlled Trial	N = 151	35 (Rituximab) 33 (Placebo)	46 (Rituximab) 45 (Placebo)
Rekeland et al [8]	2020	Intravenous Cyclophosphamide in Myalgic Encephalomyelitis/Chronic Fatigue Syndrome. An Open-Label Phase II Study	N = 40	33	52 (at 18 months)
Castro-Marrero [44]	2021	Effect of Dietary Coenzyme Q10 Plus NADH Supplementation on Fatigue Perception and Health-Related Quality of Life in Individuals with Myalgic Encephalomyelitis/Chronic Fatigue Syndrome: A Prospective, Randomized, Double-Blind, Placebo-Controlled Trial	N = 174 Treatment N = 72 Placebo N = 72	25 (Treatment) 28 (Placebo)	29 (Treatment) 30 (Placebo)
Gotaas et al [45]	2021	Cognitive Behavioral Therapy Improves Physical Function and Fatigue in Mild and Moderate Chronic Fatigue Syndrome: A Consecutive Randomized Controlled Trial of Standard (S) and Short Interventions (SI). (C = waiting list)	N = 236	53 (SI) 54 (S) 55 (C)	63 (SI) 71 (S) 58 (C)
Scheibenbogen et al [19]	2021	Tolerability and Efficacy of s.c. IgG Self-Treatment in ME/CFS Patients with IgG/IgG Subclass Deficiency: A Proof-of-Concept Study	N = 12	27	42

Although the SF-36 PF has major limitations for interpretation and is an imperfect measure, this item is often presented in clinical ME/CFS studies, both as a baseline characteristic and a possible measure of clinical outcome, as shown in Table 3. An additional concern is the use of an absolute change in SF-36 PF score as a response criterion, independent of the baseline score. One might argue that a 20-point increase in SF-36 PF from 10 to 30 would have a larger impact on quality of life as compared with an increase from 50 to 70.

As previously noted, in this study we made explorative efforts to identify groups of patients with larger symptom variations that would have had a significant influence on outcome measures in a clinical study. Dividing the patients into clinical severity groups, we observed that the mild group tended to increase more in SF-36 PF and steps per 24 hours during six months’ follow-up as compared to patients in the moderate and severe groups. When we combined SF-36 PF > 50 or DSQ-SF score < 55, we identified a group with the largest changes in SF-36 PF between four-week intervals during follow-up, i.e. 16.9 points among these 8 patients, compared to an increase of 3.4 points in the remaining 19 patients. Although we cannot generalize from this small observational study with no intervention, our data underline the difficulties in distinguishing fluctuations in the natural course of the disease from the true effect of an intervention. If similar natural variations in patient-reported and physical activity measures occurred during a clinical trial, they could be wrongfully interpreted as response to an intervention, and could affect conclusions on response and effect sizes. Natural variation of symptoms over time, and associations with baseline disease severity, are therefore important to have in mind when planning clinical trials, and should also be included in the interpretation and discussion of clinical trial results.

In order to reduce the impact of natural symptom variation in future studies, one option could be to include a run-in period before start of intervention, to identify individual variation of symptoms e.g. over a time period of three to six months, and make it easier to interpret any changes occurring after active intervention.

The feedback from the patients assessing the use of Fitbit trackers was generally positive. Most found participation in the study useful, and they considered the Fitbit app and activity armband easy to use. Most patients reported that the Fitbit measures gave an accurate reflection of their activity level. Half of the patients reported that using the activity armband influenced their activity level. From the consultations with patiens in previous studies we have learned that some patients use activity trackers to monitor and pace their physical activity, partly as a tool to prevent post-exertional malaise (PEM) and “crashes”. For some patients with ME/CFS, it is possible that wearing a tracker will decrease daily steps, at least in some periods, and give the opposite effect than the previously mentioned metaanalysis that showed increase in daily steps by wearing activity monitors [34].

The most important limitation of the study is the low number of participants in an observational study, too few to draw firm conclusions. The study population included more patients with mild disease than we usually include in our studies. There were little missing data both at baseline and follow-up. As discussed, the patients pointed at possible sources of error regarding Fitbit step registration, indicating that step count accuracy must be expected to vary between individuals as well as between devices. Nevertheless, the trackers can be a useful tool to monitor day to day changes for one individual, when the same technology is used over time.

## 5. Conclusion

In this study we have observed the course of 27 ME/CFS patients during 6 months’ follow-up without any intervention. It is feasible to use activity trackers for the continuous registration of steps and resting heart rate in a study with ME/CFS patients. According to feedback from patients, the Fitbit trackers were easy to use, and gave a fair reflection of their physical activity levels. The correlations between steps per day and SF-36 PF, SF-36 SF, and DSQ-SF scores were significant. SF-36 PF has been reported in many studies of ME/CFS, with large differences in baseline values reflecting inclusion of patient populations which may not be easily comparable. After exploring different combinations of PROMs, activity measures and clinical assessment, we found that the combination of lower SF36-PF and higher DSQ-SF defined patients with more stable symptoms during follow-up in this study with no intervention. The knowledge from this study could be useful for the design of study protocols and assessments of outcome measures in future interventional studies. We propose including a run-in period with activity tracking and PROMs pre-intervention to evaluate normal fluctuations of the disease in individual patients. Due to the complexity of symptoms, it is necessary to combine the activity measures with patient-reported outcome measures to assess different aspects of disease.

## Supporting information

S1 File Study protocol. (PDF)

S2 File R script for the Fitbit web api. (R)

S3 File Clinical data, PROMS and Steps during follow-up. (XLSX)

S4 File TREND statement checklist for clinical trials. (PDF)

S5 File Mean steps and resting heartrate during follow up, for all participants. (XLSX)

S6 File Steps measured with SenseWear compared to Fitbit, data for all patients. (XLSX)
