# Supplementary material for: Activity monitoring and patient-reported outcome measures in Myalgic Encephalomyelitis/Chronic Fatigue Syndrome patients
Source: PLoS One. 2022 Sep 19;17(9):e0274472. doi: 10.1371/journal.pone.0274472 (PMC9484698; doi:10.1371/journal.pone.0274472)
Supplement: S1 File — (PDF) [file pone.0274472.s001.pdf]

Protocol

PHYSICAL ACTIVITY REGISTRATION IN MYALGIC ENCEPHALOMYELITIS/ CHRONIC FATIGUE SYNDROME

A pilot study for continuous registration of physical activity using Fitbit armbands for 6 months. Assessment of feasibility and comparison with self-reported questionnaires.

Protocol code: KTS-8-2019

ClinicalTrials.gov: NCT04195815

Version	Date	Approved	Details of changes
1.0	10.06.2019	10.09.2019	
2.0	24.10.2019	22.11.2019	Changes in patient information after suggestions from the hospitals' Data Protection Officer. Added voluntary measurements of lactate in a home setting. Added symptom questionnaire DSQ-SF.
3.0	26.08.2021		Amended study end date to 31.12.2024

Signed (location, date, name)

------------------------------ ---------------------------------

------------------------------ ---------------------------------

Contents

PHYSICAL ACTIVITY REGISTRATION IN MYALGIC ENCEPHALOMYELITIS/ CHRONIC FATIGUE SYNDROME 1
ATTACHMENTS TO PROTOCOL 2
STUDY SITE AND RESPONSIBLE STAFF MEMBERS 3
Study site 3
Project manager/investigator 3
Investigators 3
Study coordinator 3
Biobank 3
PROJECT DESCRIPTION 4
Pilot study: ACTIVITY MONITORING IN MYALGIC ENCEPHALOPATHY / CHRONIC FATIGUE SYNDROME 7
Aim 7
Design 8
Inclusion criteria 8
Exclusion criteria 8
Information and assessment 8
Baseline registration 8
Data collection and data management 8
EXAMINATIONS AND REGISTRATION AT BASELINE 11
LABORATORY ANALYSES AT BASELINE 12
STUDY FOLLOW-UP 13
Biobank for biological studies 13
Adverse effects 14
Ethical considerations 14
Funding 15
Publication 15
REFERENCES 15

ATTACHMENTS TO PROTOCOL

A. Canadian diagnostic criteria for ME/CFS.
B. Participant instructions
C. Self-report form for ME/CFS symptoms at baseline
D. Self-report form for ME/CFS symptom change every two weeks
E. SF-36 (v1.2) questionnaire on health-related quality of life
F. Doctor’s registration of ME/CFS symptoms at baseline and during follow-up
G. Patient information / consent form

STUDY SITE AND RESPONSIBLE STAFF MEMBERS

Study site
The Department of oncology and medical physics at Haukeland University Hospital, Bergen, Norway

Project manager/investigator
Professor Øystein Fluge, MD, PhD
Dept. of oncology and medical physics
Haukeland University Hospital, 5021 Bergen, Norway

Investigators
Head of department, professor Olav Mella, MD, PhD
Dept. of oncology and medical physics
Haukeland University Hospital, 5021 Bergen, Norway

Senior consultant/PhD candidate Ingrid Gurvin Rekeland, MD
Dept. of oncology and medical physics
Haukeland University Hospital, 5021 Bergen, Norway

Study coordinator
Kari Sørland, RN, MSc
Dept. of oncology and medical physics
Haukeland University Hospital, 5021 Bergen, Norway

Biobank
Kine Alme, MSc
Dept. of oncology and medical physics
Haukeland University Hospital, 5021 Bergen, Norway

Kristin Risa, MSc
Dept. of oncology and medical physics
Haukeland University Hospital, 5021 Bergen, Norway

PROJECT DESCRIPTION

Myalgic Encephalomyelitis/Chronic Fatigue Syndrome (ME/CFS) is characterised by pathological exhaustion and malaise, particularly after strenuous activity («post-exertional malaise», PEM), along with cognitive symptoms such as difficulties with concentration and memory, sensory hypersensitivity, pain (usually from muscles, joints and new-onset headaches), sleep disturbances as well as a variety of symptoms from the autonomous nervous system. ME/CFS affects approximately 0.1-0.2 % using strict diagnostic criteria [1], and must be differentiated from more general fatigue, which affects a larger percentage of the population. Patients with serious ME/CFS have considerably reduced quality of life, and the condition carries great public socio-economic costs [2].

The cause of ME/CFS is unknown, and no universally accepted and effective treatment exists. A lack of reliable biomarkers means the diagnosis is mainly based on the patient's own experience of the illness. Diagnostic criteria are used to separate the ME/CFS patients from other conditions characterized by general fatigue [3].

At the oncology department at Haukeland University Hospital, we observed patients with long-lasting ME/CFS who were later diagnosed with cancer, and who experienced considerably improved ME/CFS symptoms after cancer treatment. During the last ten years, we have conducted several clinical trials in ME/CFS, investigating medical intervention with either the monoclonal anti-CD20 antibody rituximab or the cytotoxic drug cyclophosphamide.

In our two first phase II trials evaluating rituximab in ME/CFS, a randomised and placebo controlled study [4] and an open phase II trial of rituximab maintenance treatment [5], the end points were based on subjective, self-reported forms for health-related quality of life (QoL) (Short Form 36, SF-36) and symptom change every other week throughout follow-up (Fatigue score (scale 0-6), Function level (%)).

In our last two clinical trials; a multi-centre, randomised, double-blind and placebo controlled phase III trial of rituximab (RituxME) [6], and an open phase II trial of cyclophosphamide (CycloME, manuscript submitted for peer review), the study endpoints were based on the above data for QoL and symptom change through follow-up, with the addition of physical activity registration by SenseWear armbands (BodyMedia Inc., Pittsburgh, PA, USA) for five to seven consecutive days. Changes in mean steps per 24 hours were used as a secondary endpoint.

I the RituxME trial, objective activity measurements by SenseWear armband were recorded for five to seven days at baseline and again at 18 months. In the CycloME trial, activity registration was performed at baseline and repeated at 8-9 months, 11-12 months and 17-18 months. After an approved protocol amendment, we also performed activity registration after 24-30 months and after 38 to 44 months' follow-up.

The SenseWear armbands are thoroughly validated and have been used for the evaluation of physical activity in groups such as patients with rheumatoid arthritis, and were found to reliably monitor changes in activity post-intervention [7,8]. However, the armbands have certain limitations in terms of battery life and user-friendliness, and the armband has gone out of production.

The disease mechanisms behind ME/CFS are not known, and there are no established and recognised biomarkers. Most clinical trials for ME/CFS patients have used patient-reported,

subjective questionnaires as basis for endpoints and outcomes. The interpretation of such trials are thus subject to considerable limitations, where bias in patient reports could affect the study results.
Therefore, we aspire to establish better endpoints for clinical trials in ME/CFS. During our most recent clinical studies, the RituxME [6] and CycloME (Rekeland et al., manuscript submitted) trials, we have used SenseWear armband registrations as secondary endpoints. However, as SenseWear measurements were only conducted during limited time spans (five to seven days) and only at a few set time points during follow-up, the records could provide a skewed impression of the patients' actual activity level over time.

The last five years have seen rapid advances in the field of activity monitors, which are becoming increasingly accurate and reliable. There are a host of suppliers on the market (such as Fitbit, Apple, Garmin, Polar) and continuous improvements to technology and applications.

In this pilot study, we wish to evaluate the use of the activity bracelet Fitbit Charge 3 for continuous measurement of activity level through follow-up in clinical trials for ME/CFS patients.
A number of clinical studies have already validated previous generations of the Fitbit bracelet for activity monitoring in different patient groups. The search term «Fitbit» yields a total of 443 hits on the PubMed database, and 422 trials on ClinicalTrials.gov (per 10.06.19).
The overall impression is that Fitbit measurements of steps seem to have acceptable accuracy [9]. For example, a study of 30 patients with pulmonal hypertension, where Fitbit Charge watches were used for an average 136 days, showed correlations between changes in number of steps and changes in health-related QoL or a 6 minute walking test [10]. At a high activity level, reliability for movement was perceived as acceptable in a study comparing the previous model Fitbit Flex with the Actigraph monitor [11].
For «energy expenditure» and heart rate measurements at a high level of activity, data from previous Fitbit generations were more uncertain, compared with the Actigraph accelerometer and chest strap [12]. Sleep records are reported as somewhat inaccurate so far.

The reliability and accuracy of activity watches are expected to improve considerably during the years to come. Different types of data from the monitors (i.e. number of steps, distance covered, number of floors, minutes of different levels of activity, heart rate measurements, total calorie and active calorie expenditure, sleep parameters such as number of minutes in different sleep stages and waking periods per night) must be expected to display varying levels of accuracy.

We aim to conduct a pilot study for up to 30 participants with ME/CFS according to Canadian criteria [3], with continuous registration of physical activity by Fitbit Charge 3 through six months' observation. We will record steps, distance, heart rate, active minutes, calorie expenditure and sleep (deep, shallow, REM). Fitbit Charge 3 has a battery life of seven days, and is designed for 24-hour wear.

We will pay particular attention to step count, but will also analyse the above-mentioned parameters in order to assess which data are more descriptive of patients' activity level. We will include individuals with various degrees of ME/CFS: mild disease (able to partake in social activities and perhaps part-time study or work activities a few hours a week), moderate disease (mainly housebound) and severe disease (mainly bedbound). We will not include patients with very severe disease, who are completely bedbound and need help for all basic needs.

For the sake of comparison, the participants will complete the QoL questionnaire SF-36 and the symptom questionnaire DePaul Symptom Questionnaire – Short Form (DSQ-SF) every four weeks. In accordance with trial protocols for our previous clinical trials, they will also complete a self-report questionnaire for symptom change every two weeks through follow-up (fatigue score (scale 0 to 6, where 3 is unchanged from baseline) and function level in per cent according to a table of examples). In addition to continuous use of the Fitbit Charge 3, they will wear a SenseWear armband for 5 to 7 consecutive days at baseline and after three and six months. We will analyse associations between continuous Fitbit data, SenseWear data for three one-week periods and patient-reported data for symptom change and quality of life. Participants will also attend consultations with physicians at the department of Oncology at baseline and after three and six months.

When analysing study data, we will investigate which parameters from the Fitbit watches seem to reflect the patients' experience of symptoms and physical activity most accurately, with the aim to develop better objective endpoints with physical activity measurements for future clinical trials.

A challenge with Fitbit (and all other activity watches) is the involvement of a third party in the data stream (in this case, Fitbit International Ltd, Ireland). Activity data is recorded on the activity monitor and transferred via Bluetooth to a Fitbit app on the participants' mobile phone or tablet, which means the company has access to the participant's personal data.
We have held several meetings with the data protection officer and the IT security manager at Haukeland University Hospital to discuss the best ways to ensure participants' privacy and data protection. In collaboration with the data protection officer, we have conducted a data protection impact assessment (according to the General Data Protection Regulation (GDPR) art. 35) in order to assess any residual risks.
We have checked user terms and conditions for the leading manufacturers of activity watches. The Fitbit terms and conditions comply with the GDPR regulation and state specifically that users have the right to access, download, edit and delete personal data, refuse the use of data through privacy settings and limit the company's use of their data. Registration of personal data requires explicit consent, and the user can demand at any time that the company erase some or all data from their account.

As an additional patient privacy measure, we will use pseudonymisation when interacting with Fitbit, including study-specific e-mail addresses, fictive names and passwords that comply with strict IT security demands.

The project group downloads data via Fitbit's application programming interface (API) (https://dev.fitbit.com/). For each individual account, we need to log on to dev.fitbit.com with user name and password to retrieve "OAuth 2.0 Client ID" and "Client Secret", in order to generate an authorisation code for the API. A text file listing usernames, passwords and authorisation codes for each individual user will be stored on a secure area on the hospital's research server. The script to download the data will be stored in the same location. A separate master list linking user names to names of participants will be stored on a separate area with limited access (project manager and study coordinator only).

In the patient information leaflet, the participants are informed specifically about their rights to withdraw consent and request deletion of data. The leaflet also provides a summary of what study participation entails and of the user terms and conditions for the activity watch. They will also be given oral information tailored to their situation, and will have ample opportunity to ask any questions they may have. They will be informed specifically that their Fitbit account will be set up using the strictest privacy settings by the research group, and that any use of the Fitbit watch beyond what is defined in the protocol (e.g. if they choose to link to a third party app) could involve additional privacy risks for which the research group cannot be held accountable. User representatives have reviewed both the research protocol and the written patient information.

The above measures have been implemented in consultation with the hospital's IT safety manager and the data protection officer.

Pilot study: ACTIVITY MONITORING IN MYALGIC ENCEPHALOPATHY / CHRONIC FATIGUE SYNDROME

Aim

To investigate the feasibility of continuous activity registration using the Fitbit Charge 3 through six months observation.
To investigate which activity data (e.g. mean number of steps per 24 hours) which best reflect the participants' perception of activity level and symptom severity.
To investigate the associations between subjective, patient-reported questionnaires for quality of life (SF-36), symptoms (DSQ-SF) and symptom change (Function level (%), Fatigue score (0-6)) and continuous activity registration using the Fitbit Charge 3.
To investigate the associations between data from the concurrent use of SenseWear activity armbands and the Fitbit Charge 3 for five to seven consecutive days at baseline and after three and six months.

Additionally, for participants who wish to take part: To investigate the feasibility of self-measurement of blood lactate at home.

The overall purpose is to validate continuous measurements of activity level and investigate which parameters best reflect patient perception of ME/CFS, in order to improve endpoints for future clinical trials involving ME/CFS patients.

Design
Prospective observational study; pilot study with up to 30 participants.

Inclusion criteria
-Patients with ME/CFS according to Canadian criteria of 2003 [3] ***(attachment A)***.
-Disease duration of at least two years.
-Mild, Mild/moderate, Moderate, Moderate/severe or Severe ME/CFS.
-Age 18 to 65 years.
-Signed informed consent.

Exclusion criteria
-Patients with fatigue, who do not fulfil the diagnostic (Canadian) criteria for ME/CFS, or duration < 24 months.
-Vere severe degree of ME/CFS (bedridden and in need of care).
-Other pathology that may be the cause of patient symptoms.
-Severe endogenous (primary) depression.
-Lack of ability to complete study participation including follow-up.

Information and assessment
Candidates for inclusion will receive information about the study through a written patient letter/declaration of consent ***(attachment G)*** and a consultation with an investigator or study nurse – either at the clinic or over the telephone. Candidates who consent to participation will be invited for assessment at the Department of Oncology at Haukeland University Hospital. No study specific analyses or tests will be performed prior to written informed consent from the participant.

Baseline registration
Candidates will attend a clinical assessment with laboratory analyses, as well as blood sampling for biobank (subject to separate written informed consent).
Included participants will be issued with a Fitbit Charge 3 and written instructions on how to use the armband for continuous recording of physical activity. They will be informed on how activity data will be transferred from the Fitbit app to the study centre, every four weeks.
Participants will complete baseline questionnaires for patient-reported symptom scores, and they will be issued with a SenseWear armband for continuous activity monitoring for 5 to 7 days.

Data collection and data management
Data manager is study coordinator Kari Sørland.

At inclusion, each participant is registered and allocated a study ID number and a study specific e-mail for pseudonymisation towards a third party (Fitbit).

The participants will receive individual study folders. The study folder must be brought to each visit at the trial site. The folder contains (separated by partitions) a front page with contact information and a study calendar; a copy of the written patient information/consent form; simplified instructions for the setup and use of Fitbit Charge 3; a form for self-reporting of symptoms at baseline (scale 1-10); a form for self-reporting of symptom change (scale 0-6) and total function level (scale 0-100) every two weeks during follow-up; SF-36 and DSQ-SF questionnaires to be completed at 0, 1, 2, 3, 4, 5 and 6 months.

Participants will attend clinical visits with an investigator at baseline and after three and six months.
The study coordinator, investigators and biobank staff have received training in Good Clinical Practice (GCP). The study management at Haukeland University Hospital (Olav Mella, Øystein Fluge, Ingrid Gurvin Rekeland, and Kari Sørland) will have access to data from all included participants.
Paper copies of all completed questionnaires are kept at the study centre. After study completion, the patients' study files with original patent-reported questionnaires will be filed at the Dept. of Oncology at Haukeland University Hospital for 10 years following the final study report. All data files, including key files linking study ID with identifying information about the patients, will be stored on designated areas on the Haukeland University Hospital research server for the entire study period.
Participants will be characterized by demographic and clinical variables. We will record changes over time for each individual participant and for the group as a whole.
SPSS, «R» and Graphpad Prism may be used for statistical analyses.

Patient-reported symptom scores at baseline

Each participant completes a baseline assessment (after inclusion, before start of study) of specific symptoms (scale 1 to 10), and Function level scored as a percentage of a completely healthy state (defined as 100 %), according to a list of examples included in the participants' study folder ***(attachment C)***.

Patient-reported symptom change during follow-up

Each participant records patient-reported symptom change every two weeks throughout the study. Throughout the study, symptom change is compared to symptom status at baseline. Changes in symptom severity are scored on a scale of 0 to 6, where 3 = unchanged, 4 = slight improvement, 5 = moderate improvement, 6 = major improvement, 2 = slight worsening, 1 = moderate worsening, 0 = major worsening ***(attachment D)***.

A symptom score for the main category Fatigue is calculated every two weeks as the mean score for the following four items: Fatigue, Post-exertional malaise, Need for rest and Daily function.

A mean Fatigue score will be recorded for the time intervals 0 to 1 month, 1 to 2 months, 2 to 3 months, 3 to 4 months, 4 to 5 months and 5 to 6 months follow-up.

Function level

Changes in self-reported symptom scores compared to baseline will be relative, as significant change (i.e. value 6, scale 0-6) will be perceived differently by a patient who is somewhat active and on their feet, and a patient who is seriously ill and mainly bedridden at baseline.
Therefore, the patients will estimate their total function level every two weeks, expressed as a percentage of a totally healthy, pre-ME/CFS state, (which corresponds to 100 %), according to the list of examples in the patient folder ***(attachment C).***
We will record the mean function level for the last three months before inclusion (baseline value) and for the time intervals 0 to 1 month, 1 to 2 months, 2 to 3 months, 3 to 4 months, 4 to 5 months and 5 to 6 months follow-up.
Similar patient-report questionnaires have been used in the KTS-1-2008 [4] and KTS-2-2010 [5] trials, in the phase III RituxME trial [6] and the phase II CycloME trial (Rekeland et al, in prep).

SF-36 questionnaire on health-related QoL, DSQ-SF on ME/CFS symptoms

The Short Form 36 (SF-36) questionnaire on health-related QoL and the DePaul Symptom Questionnaire – Short Form (DSQ-SF) will be used for patient-reported measures, to be completed at baseline and at 1, 2, 3, 4, 5 and 6 months ***(attachment E)***. Completed forms are collected at clinical visits at 3 and 6 months.

SF-36 v1.2 is a generic (diagnosis-independent) form, which is widely evaluated [13,14]. We use a Norwegian validated translation [15]. In the SF-36 questionnaire for follow-up, question 2: "Compared to one year ago, how would you rate your health in general now?" has been replaced with: "Compared to before the start of the study, how would you rate your health in general now?" The SF-36 is analysed using a standardized SPSS syntax file, where the results for "Physical health summary score" and "Mental health summary score" are interpreted using norm-based scoring (population mean = 50), and the results for the eight SF-36 subdimensions can be expressed either as "raw scores" (scale 0-100) or as norm-based scores (US 1998).

The SF-36 ”Physical health summary score" (norm-based) and the SF-36 subdimension ”Physical Function" expressed as a raw score (scale 0-100) will be recorded at 0, 1, 2, 3, 4, 5 and 6 months.

The DSQ-SF is the short version of the DePaul Symptom Questionnaire, a comprehensive and well-validated questionnaire for assessment of ME/CFS, which was considered less suited for recording symptom change over time. The Short Form consists of 14 questions regarding selected and characteristic symptoms of ME/CFS. This questionnaire was recently developed and published in 2019, and has therefore not been used in our previous studies. We wish to assess the DSQ-SF for user-friendliness and compare data from the DSQ-SF to the SF-36 and patient-report questionnaires, which we have used in previous studies.

Clinical registration at baseline and through follow-up
At baseline, the investigator will assess ME/CFS severity as Mild, Mild to Moderate, Moderate, Moderate to Severe, or Severe (***attachment F***). Patients with very severe degree of ME/CFS are not included in the study.
The investigator will assess and record symptom severity for selected symptoms at baseline (scale 1 to 10). Assessment of symptom change (scale 0 to 6) will be performed at clinical visits after 3 and 6 months ***(attachment F)***.
Concomitant medications and dietary supplements are recorded.
Any discomfort or difficulties involved in the use of the Fitbit armband will be recorded. Each visit must be documented in the patient's electronic journal.

SenseWear armbands for monitoring of activity level
Physical activity level measurements by a SenseWear armband for 5 to 7 consecutive days will be performed after inclusion and consent. The measurements are repeated after approx. Three and six months.
SenseWear armbands will be issued to the patient in person or by mail from the study centre and must be returned after 7 days in an enclosed return envelope. SenseWear data will be downloaded and analysed at the Dept. of Oncology.

The SenseWear armbands record mean number of steps per 24 hours, maximum number of steps per 24 hours, total energy expenditure, active energy expenditure, mean METs (metabolic equivalents of tasks), duration of different levels of physical activity (expressed as sedentary slight activity <3.5 METs, moderate activity 3.5-6.0 METs, vigorous activity >6,0 METs), total duration of physical activity (>3,5 METs), time lying down, duration of sleep, duration on-body per 24 hours.

Analyses
After completed 6 months study participation, we will compare data from Fitbit Charge 3 and SenseWear armbands. Changes in activity measures from Fitbit armbands will be compared to changes in subjective, patient-reported data from SF-36 Physical Function (SF36-PF), DSQ-SF, Fatigue score and function level.
Our main focus will be on mean steps per 24 hours, but all variables from activity monitors will be analysed exploratively.
The aim is to establish better objective endpoints based on physical activity monitoring for future clinical trials for ME/CFS patients.
Project completion date is planned at December 31, 2024.

EXAMINATIONS AND REGISTRATION AT BASELINE

Participants will attend the outpatient clinic at the Dept. of Oncology at Haukeland University Hospital for baseline assessments (clinical assessment, laboratory tests and biobank samples (optional))

Clinical assessment

The assessing physician/investigator will check inclusion and exclusion criteria, and decide whether there is any need for supplementary work-up.
The assessment will involve exclusion of other medical conditions that may cause considerable fatigue such as hypothyreosis, adrenal insufficiency, malignity, chronic infections, lung disease, angina pectoris, heart failure, kidney failure, liver disease, other neurological diseases (multiple sclerosis, brain tumours or cerebrovascular disease), endogenous depression or other psychiatric conditions associated with fatigue.

-The doctor will assess ME/CFS symptomatology and record the severity of the symptoms (Mild, Mild to Moderate, Moderate, Moderate to Severe, Severe) on the relevant form.
-The participant will complete questionnaire for health-related QoL, SF-36 and symptoms, DSQ-SF.
-The participant will complete patient-reported questionnaire for baseline symptoms (scale 1 to 10) and total function level (0 to 100 %).

LABORATORY ANALYSES AT BASELINE

Any blood analyses featured under Immunology, Endocrinology or Microbiology (see below) which have already been performed over the last six months, need not be repeated at baseline.

General laboratory analyses

-Hb, ESR, WBC differential, Platelet count, MCV.
-Ferritin, Fe, TIBC, Vitamin B12, Folate, Na, K, Ca, Mg, Phosphate, Glucose.
-Creatinine, Urea, Urate, Total Cholesterol, HDL Cholesterol, 25-hydroxy-vitamin D.
-ALT, ALP, GGT, Bilirubin.
-CRP, Albumin, Total Protein, INR.
-HCG for women of childbearing age.
-Urine dipstick test.

Immunology

-Serum Protein Electrophoresis, Quantitative Immunoglobulins with IgG, IgG subclasses, IgM, IgA.
-Immunophenotyping of mononuclear cells in peripheral blood (lymphocyte quantification).
-tTGA (Celiac Disease Test), Antinuclear Antibody Test, anti-CCP, Thyroid Antibodies (Anti-TPO), Cardiolipin antibodies.

Endocrinology

-FT4, TSH, Prolactin, Cortisol/ACTH.

Microbiology

-Serology for EBV, CMV, Parvovirus B19, Borrelia, HIV, Hepatitis serology (HBV, HCV).

Blood samples for biobank (subject to separate consent)

SenseWear activity armbands at baseline

SenseWear armbands will be issued to the patient in person or by mail from the study centre, along with instructions to wear the armband for seven consecutive days and return it thereafter in an enclosed, prepaid return envelope.

Lactate measurements in a home setting (optional)

A hand-held, validated lactate analyser, Lactate Scout 4, will be sent by mail (with a letter of instructions, registration form, disposable lancets and a prepaid return envelope for the Lactate Scout device) twice during the study to all participants who consent to lactate measurements. Participants are instructed to measure their own blood lactate (by a pinprick to the finger) every morning while still in bed, and one hour after getting up. They are also instructed to measure their lactate levels following any strenuous activities that may cause a symptom flare/PEM, and note on the registration form which activity they had performed.

STUDY FOLLOW-UP

Participants are requested to wear the Fitbit Charge 3 continuously for 6 months (with the exception of necessary pauses for recharging).
Activity data from Fitbit are downloaded automatically via Fitbit's application programming interface (API).

Patients complete questionnaires on symptom change (scale 0 to 6) and function level (%) every two weeks throughout the study.
Participants complete SF-36 questionnaire on health-related QoL and DSQ-SF on ME/CFS symptoms every four weeks.
Participants perform SenseWear activity registration after three and six months. Armbands are returned in pre-paid envelopes after seven consecutive days of registration.
Participants who have opted for lactate measurements at home will perform these every day for one week, twice during the study.

Participants will attend the outpatient clinic at the Dept. of Oncology after three and six months (consultation with investigator, collection of questionnaires, laboratory tests and biobank samples (optional)).

Biobank for biological studies

Patients who have been included in previous clinical studies on ME/CFS at the Dept. of Oncology, HUH, have donated blood samples at baseline and through follow-up for biobank storage. Our study-specific biobanks were in August 2018 included in a new, general ME/CFS research biobank («Pathomechanisms in ME/CFS», responsible manager: Professor Olav Mella).

Patients will be invited to donate biological material (blood samples) to this biobank at baseline and after three and six months. They will receive a separate information letter and consent form for biobank sampling.
Biological material donated to the biobank will be used for biological studies on the pathogenesis of ME/CFS. More knowledge of the symptom mechanisms is required, and there is a great need for a specific and sensitive biomarker.

Adverse effects

This study is a prospective observational study with no medical intervention. The participants will use the activity armband continuously through 6 months follow-up. Adverse effects of using the armband itself are unlikely. Some participants may find the registration of their activity levels stressful.
The study management is responsible for following up on any discomfort or difficulties arising from the activity monitoring during the study.

Ethical considerations

ME/CFS is a serious condition with considerable suffering for the patient and a great strain on families and caretakers. No standard, established medical treatment exists. The disease affects many young people, with an estimated prevalence of approx. 0.1-0.2% of the population; at least 5,000 to 10,000 patients in Norway, and approx. 10,000,000 patients worldwide.

When performing clinical intervention trials in ME/CFS, researchers are faced with a lack of recognized biomarkers and pathomechanisms, which means that endpoints are largely based on subjective patient reports. Data from clinical intervention trials can thus be difficult to interpret. There is a need to establish better endpoints for clinical studies, and objective and continuous measurements of physical activity throughout a trial may serve as such an endpoint.

Use of an activity monitor such as Fitbit Charge 3 involves possible challenges concerning data protection. In collaboration with the hospital's data protection officer, we have therefore conducted a data protection impact assessment (DPIA) according to the GDPR art. 35.
We found that the Fitbit terms and conditions appear to comply with GDPR; users have the right to access, download, edit and delete personal data, refuse the use of data through privacy settings and limit the company's use of their data. Registration of personal data requires explicit consent, and the user can demand at any time that the company erase some or all data from their account.
As an additional patient privacy measure, we will use pseudonymisation when interacting with Fitbit. Central download of data via the Fitbit API requires that project staff must have access to the participant's study-specific Fitbit account password. Thorough information about data protection issues before patient consent and inclusion in the study is essential.

The data protection officer concluded that with the described measures in place, there is little risk involved, and has given a careful recommendation for the conduct of this study. However, if researchers wish to proceed using Fitbit in larger studies based on the experiences in this pilot

study, he recommends a prior consultation with the supervisory authority in order to shed light on any principal matters of concern regarding legal responsibilities and data protection.

Funding

The study is investigator initiated. There is no external sponsor. The ME/CFS research group at the Dept. of Oncology, HUH, receives support from the Kavli Trust, and this study is part funded by the Norwegian ME Association.

Publication

Study results will be published in a reputable medical journal. Co-authorship and order of authors will comply with the Vancouver guidelines.
The study will be registered in ClinicalTrials.gov before study start date.
A final report will be submitted to REC.

REFERENCES

1. Nacul LC, Lacerda EM, Pheby D, Campion P, Molokhia M, Fayyaz S, et al. (2011) Prevalence of myalgic encephalomyelitis/chronic fatigue syndrome (ME/CFS) in three regions of England: a repeated cross-sectional study in primary care. BMC Med 9: 91.
2. Institute of Medicine (2015) The National Academies Collection: Reports funded by National Institutes of Health. Beyond Myalgic Encephalomyelitis/Chronic Fatigue Syndrome: Redefining an Illness. Washington (DC): National Academies Press (US).
3. Carruthers BM, Jain AK, De Meirleir KL, Peterson DL, Klimas NG, Lerner AM, et al. (2003) Myalgic encephalomyelitis/ chronic fatigue syndrome: clinical working case definition, diagnostic and treatment protocols. J Chronic Fatigue Syndr 11: 7-36.
4. Fluge O, Bruland O, Risa K, Storstein A, Kristoffersen EK, Sapkota D, et al. (2011) Benefit from B-Lymphocyte Depletion Using the Anti-CD20 Antibody Rituximab in Chronic Fatigue Syndrome. A Double-Blind and Placebo-Controlled Study. PLoS One 6: e26358.
5. Fluge O, Risa K, Lunde S, Alme K, Rekeland IG, Sapkota D, et al. (2015) B-Lymphocyte Depletion in Myalgic Encephalopathy/ Chronic Fatigue Syndrome. An Open-Label Phase II Study with Rituximab Maintenance Treatment. PLoS One 10: e0129898.
6. Fluge O, Rekeland IG, Lien K, Thurmer H, Borchgrevink PC, Schafer C, et al. (2019) B-Lymphocyte Depletion in Patients With Myalgic Encephalomyelitis/Chronic Fatigue Syndrome: A Randomized, Double-Blind, Placebo-Controlled Trial. Ann Intern Med.
7. Almeida GJ, Wasko MC, Jeong K, Moore CG, Piva SR (2011) Physical activity measured by the SenseWear Armband in women with rheumatoid arthritis. Phys Ther 91: 1367-1376.
8. Scheers T, Philippaerts R, Lefevre J (2012) Variability in physical activity patterns as measured by the SenseWear Armband: how many days are needed? Eur J Appl Physiol 112: 1653-1662.
9. Feehan LM, Geldman J, Sayre EC, Park C, Ezzat AM, Yoo JY, et al. (2018) Accuracy of Fitbit Devices: Systematic Review and Narrative Syntheses of Quantitative Data. JMIR Mhealth Uhealth 6: e10527.

10. Sehgal S, Chowdhury A, Rabih F, Gadre A, Park MM, Li M, et al. (2019) Counting Steps: A New Way to Monitor Patients with Pulmonary Arterial Hypertension. Lung.
11. Rowe VT, Neville M (2019) Measuring Reliability of Movement With Accelerometry: Fitbit((R)) Versus ActiGraph((R)). Am J Occup Ther 73: 7302205150p7302205151-7302205150p7302205156.
12. Pope ZC, Lee JE, Zeng N, Gao Z (2019) Validation of Four Smartwatches in Energy Expenditure and Heart Rate Assessment During Exergaming. Games Health J.
13. Ware JE, Jr., Sherbourne CD (1992) The MOS 36-item short-form health survey (SF-36). I. Conceptual framework and item selection. Med Care 30: 473-483.
14. Myers C, Wilks D (1999) Comparison of Euroqol EQ-5D and SF-36 in patients with chronic fatigue syndrome. Qual Life Res 8: 9-16.
15. Loge JH, Kaasa S, Hjermstad MJ, Kvien TK (1998) Translation and performance of the Norwegian SF-36 Health Survey in patients with rheumatoid arthritis. I. Data quality, scaling assumptions, reliability, and construct validity. J Clin Epidemiol 51: 1069-1076.
